# Phytochemical Profile and Antibacterial and Antioxidant Activities of Medicinal Plants Used by Aboriginal People of New South Wales, Australia

**DOI:** 10.1155/2016/4683059

**Published:** 2016-08-02

**Authors:** Kaisarun Akter, Emma C. Barnes, Joseph J. Brophy, David Harrington, Yaegl Community Elders, Subramanyam R. Vemulpad, Joanne F. Jamie

**Affiliations:** ^1^Indigenous Bioresources Research Group, Faculty of Science and Engineering, Macquarie University, North Ryde, Sydney, NSW 2109, Australia; ^2^School of Chemistry, Faculty of Science, University of New South Wales, Sydney, NSW 2052, Australia; ^3^Yaegl Local Aboriginal Land Council, Maclean, NSW 2463, Australia

## Abstract

Aboriginal people of Australia possess a rich knowledge on the use of medicinal plants for the treatment of sores, wounds, and skin infections, ailments which impose a high global disease burden and require effective treatments. The antibacterial and antioxidant activities and phytochemical contents of extracts, obtained from eight medicinal plants used by Aboriginal people of New South Wales, Australia, for the treatment of skin related ailments, were assessed to add value to and provide an evidence-base for their traditional uses. Extracts of* Acacia implexa*,* Acacia falcata*,* Cassytha glabella*,* Eucalyptus haemastoma*,* Smilax glyciphylla*,* Sterculia quadrifida*, and* Syncarpia glomulifera* were evaluated. All extracts except that of* S. quadrifida* showed activity against sensitive and multidrug resistant strains of* Staphylococcus aureus* with minimum inhibitory concentration values ranging from 7.81 to 1000 *μ*g/mL. The sap of* E. haemastoma* and bark of* A. implexa* possessed high total phenolic contents (TPC) and strong DPPH radical scavenging abilities. A positive correlation was observed between TPC and free radical scavenging ability. GC-MS analysis of the* n*-hexane extract of* S. glomulifera* identified known antimicrobial compounds. Together, these results support the traditional uses of the examined plants for the treatment of skin related ailments and infections by Aboriginal people of New South Wales, Australia.

## 1. Introduction

The Aboriginal people of Australia have over 40,000 years of knowledge of flora and fauna as sources of food, healing agents, and other resources [1]. Numerous plant species have been utilised as traditional medicines by Australian Aboriginal people [2], in particular for the topical treatment of sores, wounds, and skin infections, ailments which are especially common in Aboriginal communities [3]. For example, a retrospective review of the medical records of 99 children attending a primary healthcare centre in a remote area of the East Arnhem region in the Northern Territory of Australia found that by one year of age, 68% and 82% of the children had presented with their first case of scabies or streptococcal pyoderma (impetigo), respectively [4]. The use of plants for the treatment of such ailments indicates that they may provide extracts or pure compounds with antimicrobial or wound healing properties. However, to date, only a limited number of these plants have been investigated for their biological activities and/or chemical constituents [2, 3].

Problems associated with skin related infectious diseases and chronic wounds are not limited to Indigenous communities but are serious global threats [5]. It is well known that infection rates have increased and antibiotic resistance has become a growing therapeutic problem [6, 7]. In combination with bacteria being one of the most important factors responsible for skin infection and delayed wound healing [8], low antioxidant levels are also associated with such diseases [9].

As part of a collaborative research program initiated upon the request of Yaegl Aboriginal people of Northern New South Wales (NSW) to help with conserving, analysing, and developing their medicinal knowledge for ecotourism and healthcare, we have ethnobotanically documented thirty-two Yaegl medicinal plants [10] and conducted preliminary biological and phytochemical studies [11, 12]. To extend this research program, we conducted a literature review in 2012 of 128 plants used as traditional medicines across NSW with regard to their distribution and habitat, documented traditional use, biological activity, and phytochemistry [3]. This review identified significant scope for further biological and chemical investigations of medicinal plants of NSW to add to the growing understanding of this resource. It also highlighted the paucity of community specific details in the published literature.

In recognition of the potential of traditional medicines for topical treatment of skin related ailments, a further literature review of medicinal plants documented in the Yaegl study [10] and NSW review [3] for these applications was conducted. This identified three plants,* Hibbertia scandens* (leaves),* Smilax glyciphylla* (leaves), and* Syncarpia glomulifera* (leaves), used by the Yaegl Aboriginal community, and five NSW plants, namely,* Acacia falcata* (bark),* Acacia implexa* (bark),* Cassytha glabella* (whole plant),* Eucalyptus haemastoma* (sap), and* Sterculia quadrifida* (leaves), for which limited or no biological and/or phytochemical studies had been undertaken.* Acacia implexa* (bark),* Acacia falcata* (bark),* Eucalyptus haemastoma* (sap), and* Sterculia quadrifida* (leaves) are reported to be used for the treatment of sores and skin complaints [3, 13],* Hibbertia scandens* (leaves) is used for the treatment of sores and rashes [10],* Cassytha glabella* (whole plant) is used for bathing (topically) to relieve pain and* Smilax glyciphylla* (leaves) to clear skin problems, aches, and pains [3, 10, 13], and sap and ash from the leaves of* Syncarpia glomulifera* are used as an antiseptic [11] (Table 1).* C. glabella *has been found to be a source of quercetin and anthocyanins [14], but no biological studies have been undertaken on this plant.* S. glyciphylla* possesses antioxidant activity and phenolic compounds have been isolated from its leaves [15].* S. quadrifida* is reported to have moderate antifungal activity but there were no reports for phytochemical studies [16]. An antibacterial triterpenoid was isolated from the bark of* S. glomulifera* [17], as well as eucalyptin and 8-desmethyleucalyptin from its leaf wax [18]. The identification of essential oils from the leaves of* S. glomulifera* has also been undertaken [19]; however, there are no reports on the biological activity of extracts of its leaves.* A. falcata*,* A. implexa*,* H. scandens*, and* E. haemastoma* have had no reports for either biological or phytochemical studies.

In this study, 70% aqueous ethanolic extracts were prepared from the selected plants. The antibacterial activities of these extracts were determined using the MTT microdilution assay method and antioxidant activity by DPPH free radical scavenging, ABTS radical scavenging activity, and ferric reducing antioxidant power (FRAP) assay methods. Qualitative phytochemical screening and the quantification of the total phenolic, flavonoid, and tannin contents of the extracts were also undertaken. Furthermore, the* n*-hexane extract of* S. glomulifera* was chosen for gas chromatography-mass spectroscopy (GC-MS) analysis, which led to the identification of several known antimicrobial and antioxidant compounds.

## 2. Materials and Methods

### 2.1. Ethics

The research with the Yaegl Aboriginal Elders was approved by the Human Research Ethics Committee at Macquarie University (HE27 JUL2007-R05356 and 5201200763). It was conducted under the framework of best ethical practice, working in partnership with Indigenous people [62], and was governed by a cooperative research agreement with the Yaegl Community [63].

### 2.2. Collection of Plant Material

The leaves of* Syncarpia glomulifera*,* Hibbertia scandens*, and* Smilax glyciphylla*, bark of* Acacia implexa* and* Acacia falcata*, sap of* Eucalyptus haemastoma,* and whole plant of* Cassytha glabella* were collected and identified by plant taxonomist David Harrington. The leaves of* Sterculia quadrifida* were collected by botanist Robert Johnstone and identified by plant taxonomist Alison Downing. The plant samples of* A. implexa* and* A. falcata* were collected from Mulgoa, NSW; the samples of* C. glabella* and* S. glyciphylla* from Macquarie University's NSW Ecology Reserve; the samples of* E*.* haemastoma*,* H*.* scandens*, and* S. glomulifera* from Macquarie University's gardens; and* S. quadrifida* from Cudgen Nature Reserve, North Coast NSW. The GPS locations of the collection sites were recorded. Voucher specimens were deposited within the IBRG Herbarium, registered with the Index Herbariorum, New York, except for* S. quadrifida* which was lodged with the Macquarie University Herbarium. The collected plant materials (except the sap of* E. haemastoma*) were thoroughly washed under running tap water and air-dried at room temperature. The dried plant materials were ground into a fine powder using a coffee grinder. The hardened sap of* E. haemastoma* was collected by scraping it from the trunk of the tree; the sap was then directly extracted with solvent.

### 2.3. Preparation of Extracts

The powdered plant samples of* A. implexa*,* H. scandens*,* S. quadrifida* and* S. glomulifera* (0.7 L × 3, 24 h intervals),* A. falcata* (0.6 L × 3, 24 h intervals),* C. glabella* and* S. glyciphylla* (0.5 L × 3, 24 h intervals), and sap of* E. haemastoma* (0.2 L × 3, 24 h intervals) were each extracted with 70% aqueous ethanol at room temperature with occasional shaking (for plant sample amounts see Table 1). The extracts were filtered under vacuum through Whatman filter paper No. 1; then the solvent removed by evaporation using a Buchi rotary evaporator at 38°C before the crude samples were freeze-dried on a CHRIST alpha 1–4 LD plus (UK) freeze dryer. The quantities of the crude extracts obtained are given in Table 1.

### 2.4. Chemicals

All chemicals were of the highest purity (≥99.0%). Ferric chloride, Dragendorff's reagent, magnesium metal strips, gallic acid, ascorbic acid, catechin, Folin-Ciocalteu reagent, sodium carbonate, vanillin, aluminium chloride, phosphate buffer, 2,2-diphenyl-1-picrylhydrazyl (DPPH), 2,2′-azinobis-(3-ethylbenzothiazoline-6-sulfonic acid) (ABTS), 2,4,6-tripyridyl-s-triazine (TPTZ), 6-hydroxy-2,5,7,8-tetramethylchroman-2-carboxylic acid (Trolox), potassium persulfate, and ferric trichloride hexahydrate (FeCl_3_·6H_2_O) were all purchased from Sigma Aldrich, USA. Hydrochloric acid (HCl), methanol, chloroform, 98% sulfuric acid, and glacial acetic acid were all analytical grade and purchased from Merck, Germany.

### 2.5. Phytochemical Analysis

Phytochemical screenings for alkaloids, flavonoids, steroids, terpenoids, tannins, saponins, and anthraquinones were conducted in accordance with published methods [64–66]. For alkaloids, 0.02 g of extract was stirred with 2 mL of 1% HCl on a steam bath and then filtered. A few drops of Dragendorff's reagent was used to treat 1 mL of filtrate. An orange precipitate indicated the presence of alkaloids. For flavonoids, 0.02 g of extract was dissolved in 1 mL of methanol. A chip of magnesium metal was added to the solution followed by the addition of a few drops of 11.6 M HCl. The occurrence of a magenta colour indicated the presence of flavonoids. For steroids, 0.02 g of extract was dissolved in 2 mL of chloroform and filtered (using Whatman No. 1 filter paper). 98% H_2_SO_4_ was carefully added to the filtrate. A reddish brown colour at the interface indicated the presence of steroids. For terpenoids, 0.02 g of extract was dissolved in 2 mL of methanol and filtered. Acetic anhydride (1 mL) was added to the filtrate and then 2 mL of concentrated H_2_SO_4_ was added carefully to the side of the tube. Formation of a reddish brown colour at the interface indicated the presence of terpenoids. For saponins, the frothing test was used. About 0.5 g of extract was mixed with 15 mL of Milli-Q water and shaken vigorously for 5 minutes. The formation of a stable froth indicated the presence of saponins. For tannins, 0.02 g of extract was dissolved in 2 mL of Milli-Q water and filtered. A few drops of 1% ferric chloride solution were added to the filtrate. Formation of a blue colour indicated the presence of tannins. Anthraquinone glycosides were detected using the Borntrager's test after hydrolysis of the extract with 10% hydrochloric acid. Chloroform was added to the hydrolysate and the contents were shaken and treated with 10% ammonia solution. The development of a pink colour indicated the presence of anthraquinone glycosides [67].

### 2.6. Total Phenolic Content of Extracts

The total phenolic content was determined using Folin-Ciocalteu reagent as reported by Muanda et al. [68] with slight modification. The samples were prepared at a concentration of 1.25 mg/mL in methanol. To 250 *μ*L of extract (1.25 mg/mL in methanol), 3.5 mL of distilled water and 250 *μ*L of Folin-Ciocalteu reagent were added and the solution was allowed to stand for 5 min. Next, 1.0 mL of 20% Na_2_CO_3_ solution was added to the mixture and the solution was left at room temperature for 1 h. Absorbance at 735 nm was read on a spectrophotometer as was that of a blank containing methanol. The phenolic content was calculated as gallic acid equivalent (GAE) by comparison with a calibration curve of gallic acid standard solutions (10–100 *μ*g/mL) and was expressed as mg gallic acid equivalent per gram of dry extract. Data were reported as mean ± SD for three replicates.

### 2.7. Total Flavonoid Content of Extracts

Total flavonoid content was determined according to the aluminium chloride colorimetric assay with slight modification [68]. The samples were prepared at a concentration of 3.35 mg/mL in methanol. At time of 0 min, 250 *μ*L of standard solution or extract was mixed with 1 mL of Milli-Q water and 75 *μ*L of 5% NaNO_2_. After 5 min, 75 *μ*L of AlCl_3_ (10%) was added to the solution and after 1 min, 500 *μ*L of NaOH (1 M) was added to the solution. Then the total solution was made up to 2.5 mL by adding Milli-Q water and mixed thoroughly. Absorbance of the mixture, pink in colour, was determined at 510 nm versus the prepared blank. The total flavonoid content was calculated as catechin equivalent by comparison to a calibration curve of catechin standard solutions (10–100 *μ*g/mL) and was expressed as mg catechin equivalent per gram of dry weight. Samples were analysed in three replications.

### 2.8. Total Condensed Tannin Content of Extracts

Total condensed tannin content was determined by the method described by Michel et al. [69] with slight modification. The samples were prepared at a concentration of 1.25 mg/mL in methanol. Sample solution (50 *μ*L) was mixed with 3 mL of 4% vanillin in methanol followed by the addition of 1.5 mL of 11.6 M HCl. The well mixed solution was allowed to stand for 15 min and absorbance was measured at 500 nm against a blank. The total condensed tannin content was calculated as catechin equivalent after comparison with a calibration curve of catechin standard solutions (10–100 *μ*g/mL) and was expressed as mg catechin equivalent per gram of dry extracts. Samples were analysed in three replications.

### 2.9. *In Vitro* Antioxidant Assays

#### 2.9.1. 2,2-Diphenyl-1-picrylhydrazyl (DPPH) Assay

The antioxidant activities of the plant extracts were determined using the DPPH radical scavenging protocol described by Liu et al. [70]. The solutions of extracts were prepared at different concentrations (6.75–100 *μ*g/mL) in methanol. DPPH solution (50 *μ*L and 1 mM) in methanol was mixed with 200 *μ*L of sample solution and the solution mixed well by shaking before being left standing at room temperature for 30 min in the dark. The absorbance was measured at 517 nm against the blank (methanol). Ascorbic acid at the same concentrations was used as the standard. All measurements were done in triplicate. The scavenging ability of the extracts was calculated using the following equation:

(1) $$\begin{array}{c} \text{Inhibition}\left(\;\%\;\right)=\frac{\left[\left({\text{Abs}}_{\text{control}}-{\text{Abs}}_{\text{sample}}\right)\right]}{{\text{Abs}}_{\text{control}}}\times 100. \end{array}$$

From a plot of concentration against percentage of inhibition, a linear regression analysis was performed to determine the IC_50_ value (the extract concentration that could scavenge 50% of the DPPH radicals).

#### 2.9.2. ABTS Radical Cation Scavenging Activity Assay

The ABTS assay method was used as directed by Adedapo et al. [71]. A stock solution was prepared by mixing 7 mM ABTS^•+^ solution in water and 2.4 mM potassium persulfate solution in water in equal volumes and allowing the mixture to react for 12–16 h at room temperature in the dark so that it reached a stable oxidative state. The working solution was then prepared by diluting with methanol to an initial absorbance of 0.700 ± 0.020 (Abs_control_) at 734 nm. The solution was prepared fresh for each analysis. The solutions of extracts were prepared at different concentrations (6.75–100 *μ*g/mL) in methanol. Then 1 mL of sample solution was mixed with 1 mL of ABTS^•+^ solution and the absorbance was measured at 734 nm after 7 min against methanol as the blank. All measurements were done in triplicate. Trolox was used as a positive control. The percentage of scavenging inhibition capacity of ABTS^•+^ of the extract was calculated using the following formula:

(2) $$\begin{array}{c} \text{Inhibition}\left(\;\%\;\right)=\frac{\left[\left({\text{Abs}}_{\text{control}}-{\text{Abs}}_{\text{sample}}\right)\right]}{\left({\text{Abs}}_{\text{control}}\right)}\times 100. \end{array}$$

IC_50_ values of the plant extracts were also determined for ABTS^•+^.

#### 2.9.3. Ferric Reducing Antioxidant Power (FRAP) Assay

The FRAP assay was carried out by following the method described by Wang et al. [72]. The FRAP reagent included 300 mM acetate buffer (3.1 g of CH_3_COONa in 16 mL glacial acetic acid), 10 mM TPTZ solution in 40 mM HCl, and 20 mM FeCl_3_·6H_2_O solution in the ratio of 10 : 1 : 1 (v/v). The solutions of extracts were prepared at a final concentration of 0.2 mg/mL in methanol. Sample solution (400 *μ*L) was mixed with 3 mL of freshly prepared FRAP solution and the solution incubated at 37°C in a water bath for 30 min. The absorbance of the samples was then measured at 593 nm. Trolox was used as a standard solution to draw the calibration curve in a concentration range of 10–100 *μ*g/mL (*Y* = 0.0056*x* + 0.0159, *R* ^2^ = 0.9993). The FRAP results were calculated as mg of Trolox equivalent per gram extract. All experiments were done in triplicate.

### 2.10. *In Vitro* Antibacterial Activity

#### 2.10.1. Microorganisms

The bacterial strains used included the Gram-positive bacterial strains, methicillin sensitive* Staphylococcus aureus* (MSSA, ATCC 29213), methicillin resistant* Staphylococcus aureus* (MRSA, ATCC BAA1026), and wild multidrug resistant* Staphylococcus aureus* (MDRSA, clinical isolate), and the Gram-negative bacterial strains,* Pseudomonas aeruginosa* (ATCC-27853) and* Escherichia coli* (ATCC 25922). All bacterial strains were kindly provided by Dr. John Merlino (Department of Microbiology, Concord Hospital, Sydney) and the work was approved by the Macquarie University Biosafety Committee (approval reference 08/06/LAB,* KAA110412BHA*).

#### 2.10.2. Culture Media

Müller Hinton II (MH) broth (Bacto Laboratories Pty Ltd., Australia) was used for the growth of all the bacterial strains. All the culture media were prepared according to the manufacturer's instructions.

#### 2.10.3. MTT Microdilution Assay

Minimum inhibitory concentrations (MIC) were determined using the MTT microdilution method as outlined by Appendino et al. with minor modification [73]. A solution of each sample (10 mg/mL) in 20% aqueous DMSO along with that of a suitable antibiotic (1 mg/mL, vancomycin for Gram-positive strains and gentamycin for Gram-negative strains) was prepared and serially diluted to give a final plant sample concentration of 2–1000 *μ*g/mL and antibiotic concentration of 0.05 to 100 *μ*g/mL in 96-well clear bottom microtitre plates. Test samples (20 *μ*L) were inoculated with 175 *μ*L of microbial culture (*A*_600_ = 0.08 diluted 100-fold in MH broth); a sterile broth control was included. A 20% DMSO control was also included and the plates were incubated at 37°C. After 18 hrs of incubation 5 *μ*L of a methanolic solution (5 mg/mL) of MTT (3-(4,5-dimethylthiazol-2-yl)-2,5-diphenyltetrazolium bromide) was added to each well and the plates further incubated at 37°C for 1 h to determine the MIC. MTT was used as an indicator of where microbial growth reduced the yellow tetrazolium bromide to a violet formazan. MIC was described as the lowest concentration of the test compounds that inhibited visible growth of the microorganisms (the last well showing no colour change of MTT from yellow to blue).

### 2.11. GC-MS Analysis of* n*-Hexane Extract of* Syncarpia glomulifera*

The 70% aqueous ethanol extract (6.0 g) of* S. glomulifera* leaves was partitioned with* n*-hexane (50 mL × 3), dichloromethane (50 mL × 3), ethyl acetate (50 mL × 3),* n*-butanol (50 mL × 3), and water (50 mL × 3) to give 950 mg, 1.0 g, 540 mg, 1.6 g, and 750 mg of each partition, respectively. The partitions were tested for their antibacterial activity against sensitive and resistant strains of* S. aureus*,* E. coli*, and* P. aeruginosa*. The* n*-hexane extract was selected for GC-MS analysis by gas liquid chromatography (GLC) and gas chromatography-mass spectrometry (GC-MS). GLC was carried out on a BP-20 column (60 m × 0.25 mm × 0.25 *μ*m). The temperature program was 50°C (5 min) to 220°C (15 min) at 3°C/min with helium as the carrier gas. The temperature of the injector and that of detector were both set at 220°C. The BP-20 column (30 m × 0.35 mm × 0.25 *μ*m), programmed from 35°C to 220°C at 3°C/min, was used for GC-MS with helium as the carrier gas and an injector temperature of 220°C for the column. Mass spectra were recorded in electron impact (EI) mode at 70 eV, scanning from 41 to 450* m/z*. Compounds were identified by their identical GC retention times and retention indices relative to* n*-alkanes and by comparison of their mass spectra with either pure standards or published spectra in the NIST GC-MS library and those in the literature [56–61].

### 2.12. Statistical Analysis

All results are expressed as means ± standard deviation. Statistical analyses were performed using Microsoft Excel. The IC_50_ values were calculated by regression analysis. Values with *p* < 0.05 and *p* < 0.01 were considered statistically significant and very significant, respectively. The experimental results were compared by paired *t*-test (two sided).

## 3. Results and Discussion

### 3.1. Phytochemical Screening

Qualitative phytochemical tests of the 70% aqueous ethanol extracts of the eight plants showed the presence of alkaloids, terpenoids, flavonoids, steroids, saponins, tannins, and anthraquinones (Table 2). These classes of phytochemicals are known to possess a variety of biological activities including antimicrobial, antioxidant, anti-inflammatory, antiplasmodial, and anticancer activities [74–84]. These findings may partially justify the traditional use of the examined plants in the treatment of wound and skin infections and free radical mediated diseases and indicate that they may serve as a source of bioactive compounds against these illnesses.

### 3.2. Total Phenol, Flavonoid, and Condensed Tannin Contents

Phenolic compounds are effective hydrogen donors, making them good antioxidants [80]. Plant derived polyphenolic flavonoids are also well known to exhibit antioxidant activity. Flavonoids reduce free radicals by quenching, upregulating, or protecting antioxidant defences and chelating radical intermediate compounds [85]. It is also reported that tannins are 15–30 times more effective in quenching peroxyradicals than simple phenolics [86].

The phenolics and polyphenols are one of the largest groups of secondary metabolites to have exhibited antimicrobial activity [87]. The site(s) and number of phenol groups are thought to be related to their relative toxicity to microorganisms, with evidence that increased hydroxylation results in increased toxicity [88]. Naturally occurring plant flavonoids have also been reported to possess antimicrobial activities [79, 89, 90]. The variation in the antibacterial activity of flavonoids is known to be related to their chemical structure, especially in regard to the number and positions of methoxy and phenolic groups within their structures [91–93]. The antimicrobial effects of tannins have also been widely recognised [94–96]. Therefore, the total phenolic, flavonoid, and condensed tannin contents of the eight plant extracts were examined to see if their traditional uses for the treatment of skin related ailments could be linked to the presence of these classes of compounds.

**Figure 1:**
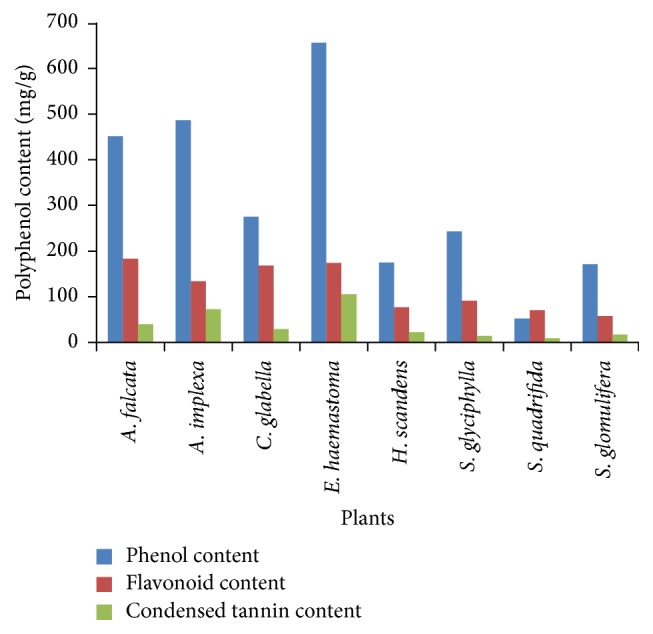
Polyphenolic contents (mg/g) of plant extracts.

The results showed that the amount of total phenolic, flavonoid, and condensed tannin contents differed significantly (*p* < 0.05) among the extracts of the tested medicinal plants (Table 3, Figure 1). The total phenolic contents were determined as mg GAE/g extract on comparison with a standard gallic acid graph. Three extracts showed very high phenolic contents (>400 mg GAE/g):* E. haemastoma*,* A. implexa*, and* A. falcata *with values of 656.2 ± 5.1, 486.7 ± 9.9, and 451.7 ± 1.3 mg GAE/g of extract, respectively.* C. glabella* and* S. glyciphylla* showed reasonable phenolic contents (>200.0 mg GAE/g) of 275.5 ± 8.6 and 243.5 ± 5.9 mg GAE/g of extract.* S. quadrifida* showed the lowest phenolic content at 52.5 ± 0.6 mg GAE/g extract. The total flavonoid content was determined as mg CE/g extract after comparison with a catechin standard graph. The highest total flavonoid content was identified for* A. falcata* at 183.3 ± 6.0 mg CE/g of extract and the lowest for* S. glomulifera* at 58.0 ± 2.2 mg CE/g of extract. The total condensed tannin content was evaluated as mg CE/g of extract after comparison with a catechin standard graph.* E. haemastoma* showed the highest condensed tannin content at 106.0 ± 5.3 mg CE/g of extract and* S. quadrifida* the lowest at 9.4 ± 2.0 mg CE/g of extract.

The results revealed that the level of phenolic compounds and condensed tannins was the highest in the 70% ethanolic extracts from the sap of* E. haemastoma* and bark of* A. falcata* and* A. implexa*. These results were significantly higher than that of the leaves of the other plants investigated.

### 3.3. *In Vitro* Antioxidant Activity

#### 3.3.1. DPPH Radical Scavenging Activity

**Figure 2:**
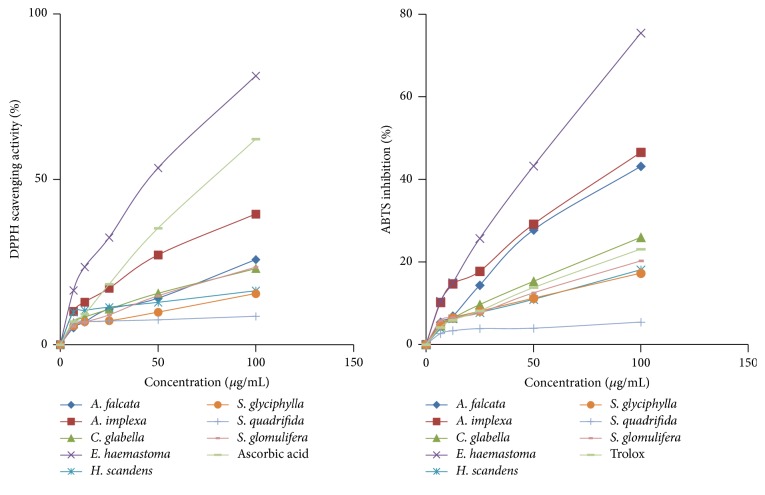
DPPH and ABTS scavenging activities of plant extracts.

The results of the free radical scavenging activity of the extracts are shown in Table 4. The dose-response curves of the DPPH radical scavenging activities of the eight plant extracts were compared with that of ascorbic acid (Figure 2). In the DPPH assay, all extracts examined except for that of* S. quadrifida* showed radical scavenging activity in a concentration dependent manner and were significantly different (*p* < 0.01). This result agreed with an earlier report by Motalleb et al. [97] that showed that the scavenging effects on the DPPH radical increase sharply with increasing concentration of the samples and standards. The highest antioxidant activity was obtained for the extract of* E. haemastoma* (IC_50_  52.0 ± 1.2 *μ*g/mL and standard ascorbic acid IC_50_  71.6 ± 1.0 *μ*g/mL).

#### 3.3.2. ABTS^•+^ Scavenging Activity

The antioxidant activities of the plant extracts towards ABTS^•+^ were also determined (Table 4, Figure 2). All extracts showed the ability to neutralise the radical cation ABTS^•+^, with significant differences at *p* < 0.01. The highest activity was obtained for the* E. haemastoma* extract with IC_50_ value of 61.7 ± 0.5 *μ*g/mL, followed by* A. implexa* and* A. falcata* with IC_50_ values of 107.1 ± 1.4 *μ*g/mL and 111.5 ± 0.9 *μ*g/mL, respectively. These extracts could be seen to be rapid and effective scavengers of the ABTS^•+^ radical (Figure 2) and their activities were comparable with that of Trolox.

#### 3.3.3. Ferric Reducing Antioxidant Power (FRAP) Assay

The FRAP assay was used to evaluate the antioxidant properties of the extracts based on their ability to reduce ferric (III) to ferrous (II). The results obtained from the extracts (Table 4) were significantly different (*p* < 0.01). For this assay it was also found that the extract of* E. haemastoma* provided the highest antioxidant activity with a FRAP value of 6189 ± 9.5 *μ*mol Trolox equivalent/g, followed by* A. implexa* and* A. falcata* with FRAP values of 2913 ± 6.8 and 1991 ± 2.7 *μ*mol Trolox equivalent/g, respectively.

Based on the results of all three assays, it can be seen that the sap extract of* E. haemastoma* and bark extracts of* A. implexa* and* A. falcata* possess the strongest free radical scavenging activities and reducing capacities of all the plant extracts analysed, indicating that they may be useful for treating free radical-related diseases. The scavenging of the ABTS^•+^ radical by the extracts was found to be higher than that for the DPPH radical. It is well known that the antioxidant activity of a plant extract largely depends on both its composition and the test system [72].

### 3.4. Correlation between the Total Phenolic and Flavonoid Contents and Antioxidant Activities

Table 5 shows the correlations (linear regression coefficients, *R* ^2^) between the total phenolic contents (TPC) and total flavonoid contents (TFC) and the antioxidant assay results for the plant extracts. All the antioxidant assay results showed very good correlation (*R* ^2^ > 0.9) with the TPC and TFC values except for that of* S. quadrifida*, which showed poor correlation between ABTS and TFC (*R* ^2^ = 0.7953). The significant correlations between the antioxidant properties and TPCs and TFCs of the extracts may indicate that the phenolic and flavonoid type compounds contained within the plant extracts are the major contributors to their antioxidant properties.

### 3.5. Antibacterial Activities

The 70% aqueous ethanol extracts of the plants were tested for their antibacterial activity using the MTT microdilution assay method against three Gram-positive (*S. aureus* (MSSA) ATCC 29213,* S. aureus* (MRSA) ATCC BAA 1026,* S. aureus* (MDRSA)) and two Gram-negative (*E. coli β*-lactamase negative ATCC 25922 and* P. aeruginosa* ATCC 27853) bacterial strains. The minimum inhibitory concentration (MIC) values for the extracts are shown in Table 6. None of the extracts showed activity against the Gram-negative bacterial strains, even at a concentration of 1 mg/mL. Seven of the eight extracts showed activity against the sensitive and/or resistant strains of* S. aureus* at MIC values of ≤1 mg/mL, except for* S. quadrifida* which did not show any activity even at a concentration of 1 mg/mL.

According to Ríos and Recio [98] extracts possessing an MIC value equalling or less than 1000 *μ*g/mL are considered to be active and worthy of further investigation. The* S. glomulifera* extract showed the greatest activity against sensitive and resistant strains of* S. aureus* with MIC values of 7.81 *μ*g/mL against all three strains, followed by* E. haemastoma* and* A. implexa* with MIC values of 62.5 *μ*g/mL and 125 *μ*g/mL, respectively, against the sensitive strain of* S. aureus*.

It is well known that phenolic compounds present in plant extracts play an important role in their antimicrobial effects [99]. Phytochemical screening of the extracts in this study showed that* E. haemastoma*,* A. implexa*, and* A. falcata* possess a high content of phenolic compounds. It has also been reported that the active constituents in* A. implexa* and* A. falcata* include tannins [99]. Therefore, it can be inferred that the antibacterial activities of these three plants may be due, at least in part, to their high phenolic and tannin contents. This is the first report of the antibacterial activities of all of these eight medicinal plants against sensitive and resistant strains of* S. aureus*. The promising antibacterial activities of the extracts provides preliminary support for the traditional uses of these plants for the treatment of skin and wound infections.

The extract of* S. glomulifera* leaves showed the highest antibacterial activity with MIC values of 7.81 *μ*g/mL against the methicillin sensitive, methicillin resistant, and multidrug resistant strains of* S. aureus*. Therefore, this extract was chosen for further investigation. The crude 70% aqueous ethanol extract was partitioned with* n*-hexane, dichloromethane, ethyl acetate,* n*-butanol, and water and the partitions tested for their antibacterial activity against sensitive and resistant strains of* S. aureus*,* E. coli*, and* P. aeruginosa* (Table 7). Among the five partitions, the* n*-hexane extract showed the greatest antibacterial activities against sensitive and resistant strains of* S. aureus* with MIC values of 7.81 *μ*g/mL against all three strains. None of the extracts showed antibacterial activity against* P. aeruginosa*, and only the* n*-hexane partition showed activity against* E. coli* at a concentration of 1 mg/mL. As the* n*-hexane extract showed the greatest activity, it was chosen for GC-MS analysis to further explore its chemical constituents.

### 3.6. GC-MS Analysis of* n*-Hexane Extract of* S. glomulifera*

GC-MS analysis of the* n*-hexane extract of* S. glomulifera* showed that it predominantly contained monoterpene hydrocarbons (*α*-phellandrene,* p*-cymene, terpinolene), oxygenated monoterpenes (terpinen-4-ol, *α*-terpineol), sesquiterpene hydrocarbons (*α*-copaene, *β*-elemene, aromadendrene, alloaromadendrene, *α*-selinene, *β*-selinene, bicyclogermacrene, and viridiflorene), and oxygenated sesquiterpenes (spathulenol, cubenol, epicubenol, cubeban-11-ol, palustrol, epiglobulol, globulol, ledol, and viridiflorol) (Table 8). These phytoconstituents are in accordance with a previous report on the chemical composition of the leaf essential oil of* S. glomulifera*, but they are present in different concentrations [19]. This could be due to seasonal variation, the different collection sites, variances in the extraction processes, or other factors [100].

*α*-Phellandrene, *α*-copaene, aromadendrene, terpinen-4-ol, *α*-terpineol, palustrol, epiglobulol, cubenol, globulol, and spathulenol have been reported to have antibacterial activity against Gram-positive bacteria [20, 28, 31–33, 42, 43, 47, 52] and the presence of these bioactive phytoconstituents could be contributing to the strong antibacterial activity of the* n*-hexane extract. Bicyclogermacrene is reported to be a major component of the antibacterial essential oil from* Zanthoxylum rhoifolium* [39]. In addition, *α*-phellandrene,* p*-cymene, terpinolene, *α*-copaene, aromadendrene, terpinen-4-ol, alloaromadendrene, *α*-terpineol, palustrol, ledol, epicubenol, globulol, viridiflorol, and spathulenol are known to possess other biological activities relevant to skin related ailments including antifungal, antioxidant, anti-inflammatory, and antiseptic activities [20, 21, 23–26, 29, 33–35, 38, 42, 45–48, 51, 54].

## 4. Conclusion

Our study has shown that extracts of* E. haemastoma*,* A. implexa*,* A. falcata*, and* S. glomulifera* contain antioxidant and antibacterial compounds. The highest* in vitro* antioxidant activity of the plant extracts was found for* E. haemastoma*, with results comparable with that of the standard compound, ascorbic acid.* S. glomulifera* and* E. haemastoma* presented the best antibacterial activities against methicillin sensitive, methicillin resistant, and multidrug resistant strains of* S. aureus*, with MIC values between 7.81 and 125 *μ*g/mL. GC-MS analysis of the* n*-hexane extract of* S. glomulifera* revealed the presence of antioxidant and antibacterial compounds. Thus, the results of this study support the use of these plants as traditional medicines for the treatment of skin related ailments including sores, wounds, and skin infections by New South Wales Aboriginal people.

## Figures and Tables

**Table 1:** Summary of plants, traditional uses, parts used, and quantities of extracts obtained.

Plant name and family	Common names^a,b^	Distribution in Australia	Traditional use	Voucher number	GPS location of plant collection	Part extracted (g)	Extract yield (g/100 g dry wt)
*A. falcata*, Fabaceae	Hickory, lignum vitae, Sally	NSW, Qld^b^	Bark used for sores and skin complaints^b,c^	IBRG00013	−33.818587, 150.614472	Bark (95.4)	20.6

*A. implexa*, Fabaceae	Black wattle, lightwood, fish wattle, broad leaf wattle, scrub wattle, hickory, hickory wattle, Sally wattle	ACT, NSW, Qld, Tas, Vic^b^	Bark used for sores and skin complaints^b,c^	IBRG00014	−33.818587, 150.614472	Bark (162.0)	11.2

*C. glabella*, Lauraceae	Devil's twine, dodder laurel, slender devil's twine, slender dodder-laurel, smooth cassytha	NSW, Qld, SA, Tas, Vic, WA^b^	Whole plant used for bathing of body to relieve pain, rheumatism, and fever^b,c^	IBRG00015	−33.769473, 151.117169	Whole plant (26.2)	11.4

*E. haemastoma*, Myrtaceae	Scribbly gum, snappy gum, white gum	NSW^b^	Sap used for cuts, sores, wounds, ulcers, and dysentery^b,c^	IBRG00011	−33.771540, 151.119465	Sap (32.6)	65.0

*H. scandens*, Dilleniaceae	Yellow vine	NSW, Qld^d^	Used to treat sores and rashes (plant part used unknown)^e^	IBRG00017	−33.773865, 151.117391	Leaves (102.0)	9.8

*S. glyciphylla*, Smilacaceae	Native sarsaparilla, sweet sarsaparilla, smooth sarsaparilla	NSW, Qld^b^	Leaves topically used to clear skin problems^b,e^, leaves and black fruits used for aches, pains, rheumatism, blood cleanser/tonic, sickness, cough, colds, congestion, and scurvy^f^	IBRG00012	−33.768539, 151.117406	Leaves (27.6)	17.0

*S. glomulifera*, Myrtaceae	Luster, red luster, turpentine, red turpentine	NSW, Qld^a^	Leaf ash and sap used as antiseptic^f^	IBRG00018, IBRG00019	−33.781832, 151.114339; −33.776060, 151.117111	Leaves (100.0)	24.0

*S. quadrifida*, Malvaceae	Kuman, orange fruited kurrajong, red fruited kurrajong, smooth seeded kurrajong, peanut tree, small flowered kurrajong	NSW, NT, Qld^b^	Leaves used to treat wounds, sores, skin complaints, sore eyes, and stings^b,c^	NSW 970302	−28.3526225, 153.564382	Leaves (80.2)	7.0

^a^Retrieved from http://bie.ala.org.au/; ^b^[3]; ^c^[13]; ^d^retrieved from http://anpsa.org.au/; ^e^[10]; ^f^[11].

ACT: Australian Capital Territory; NSW: New South Wales; NT: Northern Territory; Qld: Queensland; SA: South Australia; Tas: Tasmania; Vic: Victoria.

**Table 2:** Qualitative phytochemical screening of plant extracts.

Plant	Alkaloids	Flavonoids	Steroids	Terpenoids	Tannins	Saponins	Anthraquinones
*A. implexa*	+	+	+	+	+	+	−
*A. falcata*	−	+	+	+	+	−	+
*C. glabella*	−	+	+	+	+	−	−
*E. haemastoma*	+	+	+	+	+	+	+
*H. scandens*	−	+	+	+	+	−	−
*S. glyciphylla*	−	+	+	+	+	−	−
*S. quadrifida*	−	+	+	+	+	−	−
*S. glomulifera*	−	+	+	+	+	−	−

+ = present; − = not present.

**Table 3:** Total phenol, flavonoid, and condensed tannin contents of plant extracts.

Plant	Total phenolic content (mg GAE/g plant extract)^*∗*^	Total flavonoid content (mg CE/g plant extract)^*∗*^	Total condensed tannin content (mg CE/g plant extract)^*∗*^
*A. falcata*	451.67 ± 1.26	183.33 ± 6.04	39.86 ± 2.36
*A. implexa*	486.71 ± 9.90	133.97 ± 6.12	72.63 ± 5.03
*C. glabella*	275.52 ± 8.56	168.57 ± 0.35	29.41 ± 2.50
*E. haemastoma*	656.22 ± 5.07	172.4 ± 3.55	105.97 ± 5.29
*H. scandens*	174.66 ± 4.09	77.47 ± 3.96	21.97 ± 2.31
*S. glyciphylla*	243.47 ± 5.90	91.25 ± 4.85	14.67 ± 1.22
*S. quadrifida*	52.46 ± 0.63	70.5 ± 1.45	9.41 ± 2.04
*S. glomulifera*	171.41 ± 5.62	58.03 ± 2.15	17.41 ± 2.04

^*∗*^Results are mean ± SD from three sets of independent experiments, each set in triplicate.

**Table 4:** Antioxidant activities of plant extracts.

Plant	DPPH IC_50_ (*µ*g/mL)	ABTS IC_50_ (*µ*g/mL)	FRAP (*µ*mol Trolox/g)
*A. falcata*	217.03 ± 3.80	111.47 ± 0.88	1991.46 ± 2.73
*A. implexa*	130.20 ± 5.37	107.05 ± 1.38	2913.87 ± 6.76
*C. glabella*	255.23 ± 2.32	203.46 ± 1.25	1796.22 ± 4.58
*E. haemastoma*	51.99 ± 1.17	61.72 ± 0.53	6189.64 ± 9.45
*H. scandens*	348.69 ± 2.90	321.03 ± 3.46	1635.51 ± 5.94
*S. glyciphylla*	439.33 ± 2.05	351.46 ± 1.98	185.80 ± 5.85
*S. quadrifida*	2190.13 ± 2.16	1824.96 ± 4.26	722.41 ± 6.25
*S. glomulifera*	235.86 ± 3.50	287.98 ± 1.75	1522.11 ± 4.92

Ascorbic acid	71.58 ± 0.99		
Trolox		231.90 ± 1.76	

**Table 5:** Correlation values (*R* ^2^) between the antioxdant activities and total phenolic and total flavonoid contents of the plants extracts.

Plant	*R* ^2^ (DPPH)	*R* ^2^ (ABTS)	*R* ^2^ (FRAP)
*A. falcata*			
TPC	0.9984	0.9456	0.9844
TFC	0.9881	0.9728	0.9969

*A. implexa*			
TPC	0.9768	0.9749	0.9832
TFC	0.9157	0.9190	0.9025

*C. glabella*			
TPC	0.9695	0.9492	0.9675
TFC	0.9431	0.9167	0.9405

*E. haemastoma*			
TPC	0.9989	0.9969	0.9981
TFC	0.9737	0.9946	0.9926

*H. scandens*			
TPC	0.9987	0.9999	0.9675
TFC	0.9915	0.9857	0.9806

*S. glyciphylla*			
TPC	0.9991	0.9967	0.9977
TFC	0.9888	0.9938	0.9922

*S. glomulifera*			
TPC	0.9962	0.9939	0.9963
TFC	0.9993	0.9981	0.9993

*S. quadrifida*			
TPC	0.9961	0.9357	0.9988
TFC	0.9261	0.7953	0.9683

TPC: total phenolic content; TFC: total flavonoid content.

**Table 6:** Antibacterial activities of plant extracts.

Plant	MIC (*µ*g/mL)		
	*S. aureus* (MSSA)	*S. aureus* (MRSA)	*S. aureus* (MDRSA)
*A. falcata*	250	1000	1000
*A. implexa*	125	250	250
*C. glabella*	500	1000	1000
*E. haemastoma*	62.5	125	125
*H. scandens*	500	1000	1000
*S. glyciphylla*	1000	na	na
*S. quadrifida*	na	na	na
*S. glomulifera*	7.81	7.81	7.81

Vancomycin	0.002	0.002	0.002

na: not active at concentration of 1 mg/mL. MIC: minimum inhibitory concentration.

**Table 7:** Antibacterial activities of *Syncarpia glomulifera* partitions.

Extracts	MIC (*µ*g/mL)			
	*S. aureus* (MSSA)	*S. aureus* (MRSA)	*S. aureus* (MDRSA)	*E. coli*
*n*-Hexane	7.81	7.81	7.81	1000
Dichloromethane	31.25	31.25	125	na
Ethyl acetate	na	na	na	na
*n*-Butanol	1000	1000	1000	na
Water	1000	na	na	na

Vancomycin	0.002	0.002	0.002	NT
Gentamycin	NT	NT	NT	1.69

na: not active at concentration of 1000 *µ*g/mL. NT: not tested. MIC: minimum inhibitory concentration.

**Table 8:** GC-MS analysis of *n*-hexane extract of *S. glomulifera* on BP-20 column, phytoconstituents identified and their known biological activities.

Compounds^1^	LRI values	% of identified compounds	Known biological activities
*α*-Phellandrene	1166	0.93	Antibacterial [20], antifungal [20], antioxidant [21], larvicidal [22]
*p*-Cymene	1269	0.22	Antifungal [23–26], antioxidant [21]
Terpinolene	1282	0.08	Antioxidant [21], antiviral [27], larvicidal [22]
*α*-Copaene	1499	0.10	Antibacterial [28], antidermatophytic [29]
*β*-Elemene	1600	0.10	Anticancer [30]
Aromadendrene	1603	2.33	Antibacterial [31], antioxidant [21]
Terpinen-4-ol	1613	0.18	Antibacterial [32, 33], antifungal [33, 34], antioxidant [21], antiseptic [35], antiviral [27, 36]
Alloaromadendrene	1643	0.64	Antineoplastic [37], antioxidant [38]
Viridiflorene	1681	0.13	None found
Geranial	1685	0.92	None found
*α*-Terpineol	1696	0.41	Antibacterial [33], antifungal [33, 34], antiviral [27, 36]
*β*-Selinene	1715	0.23	None found
*α*-Selinene	1722	0.26	None found
Bicyclogermacrene	1733	0.60	Antibacterial [39]^*∗*^, antitumor [40]^*∗*^, cytotoxic [41]
Palustrol	1931	0.26	Antibacterial [42], antifungal [42], antitumor [42]
Cubeban-11-ol (cis)	2012	0.27	None found
Epiglobulol	2018	1.08	Antibacterial [43], uterus relaxant [44]
Ledol	2034	0.95	Antimicrobial [45], anti-inflammatory [45], antineoplastic [37]
Cubenol	2058	0.70	Antibacterial [28]
Cubeban-11-ol (trans)	2064	0.83	None found
Epicubenol	2070	0.36	Antifungal [46]
Globulol	2080	5.31	Antibacterial [43, 47], antifungal [47], antioxidant [48], sedative and anaesthetic [49]
Viridiflorol	2088	1.88	Acetylcholinesterase inhibitory [50], antifungal [51]
Spathulenol	2129	0.96	Antibacterial [52], anticancer [53], anti-inflammatory [54]^*∗*^, immunomodulatory [55], uterus relaxant [44]

^1^The compounds were identified by their GC retention times and linear retention indices relative to *n*-alkanes and by comparison of their mass spectra with pure standards or published literature data [56–61]. ^*∗*^Major components of essential oils with biological activities.
